# Cadmium Accumulation in Tissues of *Sarotherodon melanotheron* (Rüppel, 1852) from the Aby Lagoon System in Côte d’Ivoire

**DOI:** 10.3390/ijerph9030821

**Published:** 2012-03-08

**Authors:** Yapi Dope Armel Cyrille, Kouame Victor, Tidou Abiba Sanogo, Sawadogo Boukary, Wethe Joseph

**Affiliations:** 1 Laboratoire des Sciences de l’Environnement (LSE), Université d’Abobo-Adjamé, 02 BP 801 Abidjan 02, Côte d’Ivoire; Email: kvictor2@yahoo.fr (K.V.); atidou2000@yahoo.fr (T.A.S.); 2 Laboratoire Eau, Dépollution, Ecosystèmes et Santé (LEDES), Institut International d’Ingénierie de l’Eau et de l’Environnement, 01 BP 594 Ouagadougou 01, Burkina Faso; Email: boukary.sawadogo@2ie-edu.org (S.B.); joseph.wethe@gmail.com (W.J.)

**Keywords:** cadmium, Aby Lagoon, accumulation, *Sarotherodon melanotheron*

## Abstract

This study assessed the concentrations of cadmium in the gills, livers and muscles of a commercially important tilapia fish (*Sarotherodon melanotheron*) from Aby Lagoon in Adiaké, Côte d’Ivoire, between January and December, 2010. The organisms were grouped into two composite samples (juvenile and adult) of five individuals. Levels of cadmium were determined in tissues using Perkin-Elmer (AAnalyst 200) Atomic Absorption Spectrophotometry (AAS) after a digestion method. Fish muscle appeared to have a significantly higher tendency to accumulate cadmium (1.19–5.18 µg/g dw) while gills and livers had minimum concentrations (0.07–1.32 and 0.12–1.25 µg/g dw). This study has revealed that the concentrations of Cd in *Sarotherodon melanotheron* muscle tissue were above the maximum acceptable concentrations for human consumption, thus precautions need to be taken in order to prevent future contamination.

## 1. Introduction

Generally, heavy metal pollution in the aquatic environment is the result of industrial wastes, geochemical structure and mining activities. Under some environmental conditions, heavy metals may accumulate to a toxic concentration [1], and cause ecological damage [2]. Pollution enters fish through five main routes: via food or non-food particles, gills, oral consumption of water and the skin. The absorbed pollutants are then carried in the blood stream to either a storage point or to the liver for transformation and/or storage.

Heavy metals are present in the aquatic environment where they bioaccumulate along the food chain. For this reason, determination of chemical quality of aquatic organisms, particularly the contents of heavy metals is extremely important for human health. Therefore, the problem of food (including fish) contamination by toxic metals is receiving global attention.

Fish are often used as subjects to investigate toxic substances present in water [3], as studies have indicated that fish are able to accumulate and retain heavy metals from their environment and that accumulation of metals in tissues of fish is dependent upon exposure concentration and duration as well as other factors such as salinity, temperature hardness and metabolism of the animals [4,5].

Besides the importance of Aby Lagoon to local fisheries and also its biodiversity, this lagoon is considered as a wildlife protection area. For the past several decades, the increasing usage of heavy metals in industry has led to serious environmental pollution through effluents and wastes. The enrichment of heavy metals in Aby Lagoon has been reported in previous research papers [6,7]. In the present work, cadmium accumulation levels in fish tissues (livers, gills and muscles) of *Sarotherodon melanotheron* from Aby Lagoon were determined to assess the public health risks associated with consuming fish harvested from this area.

## 2. Experimental Section

### 2.1. Study Area

**Figure 1:**
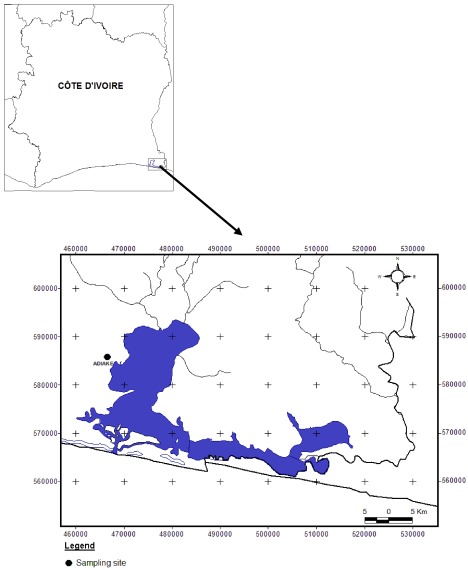
Map showing Aby lagoon and sampling site.

The study was carried out in the Côte d’Ivoire’s rural Aby Lagoon system, located between longitudes 2°51'N and 3°21'N and latitudes 5°05'W and 5°22'W. Two main tributaries (Bia and Tanoe) are escape routes from anthropogenic and mining operations within its watershed in Côte d’Ivoire and Ghana [8]. Fish samples were collected at Adiake station during the three main seasons (January to April, May to August and September to December) (Figure 1).

### 2.2. Sampling Method

*Sarotherodon melanotheron* species were caught with local fishermen’s nets seasonally and taken daily to the laboratory. Temperature and pH were also measured using a WTW pH 90; dissolved oxygen was measured using a Crison Oxy 330 and salinity was measured using a WTW 340i type conductivity meter.

### 2.3. Samples Preparation

The same day of sampling, fish were transported to the laboratory prior to analysis. Fish samples were thawed at room temperature. The organisms were grouped into two composite samples (juvenile and adult) of five individuals for each season. Their length was recorded (Table 1).

**Table 1:** Size (cm) of *Sarotherodon melanotheron* specimens.

	Dry Season (Jan.–Apr.)		Rainy Season (May–Aug.)		Flood Season (Sep.–Dec.)	
	Juvenile	Adult	Juvenile	Adult	Juvenile	Adult
**M ± sd**	15.5 ± 0.28	22.8 ± 2.02	15.25 ± 0.1	21.1 ± 0.7	15.38 ± 0.3	21.7 ± 0.8
**Min-Max**	[15.2–15.8]	[19.5–25.0]	[15.1–15.4]	[20.1–21.8]	[15.1–15.8]	[20.5–22.4]
**%CV**	2	9	1	3	2	4

M ± sd: Mean values ± standard deviation, Min: minimum, Max: maximum, %CV: Coefficient of variance

### 2.4. Preparation of Standard Solutions

Calibration standards were prepared from aqueous certified standard of cadmium (Perkin Elmer), and the acid concentration of the samples was adjusted in order to minimize the matrix effect. The concentration of all commercial stock solutions (reference standard) is 1000 ppm in order to ensure stability. Standards of 1, 2 and 3 ppm were prepared. We performed a calibration according to the requirements of Directive 2001/22/EC of the European Commission on March 8, 2001. A pure blank (control) were also prepared to check the quality of the samples. 

### 2.5. Preparation of Samples for Assaying Through Atomic Absorption Spectrophotometer (AAS)

Fishes were dissected using stainless steel scalpels. Tissues (livers, gills and muscles) were dried to constant weight at 60 °C. The grinding technique does not necessarily produce a uniform sample, but it facilitates the digestion step and increases the level of reproducibility and cross-contamination between samples is negligible. After grinding aliquots of approximately 0.2 g dried liver, 0.4 g dried gill and 0.4 g dried muscle were digested in Teflon beakers for 12 h at room temperature and then for 4 h at 100 °C with 5 mL Suprapur nitric acid (65%, Merck). The samples were cooled to room temperature and then transferred to 25 mL volumetric flasks with 2% of HNO_3_. The samples were filtered through a 0.45 µm membrane filter [9]. A Perkin Elmer (AAnalyst 200) Atomic Absorption Spectrophotometer was employed for the analysis. The assay was performed by interpolation on standard curves prepared from standard solutions.

### 2.6. Calculations

The results are expressed in µg/g dry weight or µg/g dw. To get the corrected concentration this value was multiplied by the dilution factor of the sample and divided by the initial weight of the sample before digestion [10]:

*Actual concentration of metal in sample =* *ppmR* × *dilution factor*

*ppmR* *= AAS Reading of digest*

*Dilution Factor* *= Volume of digest used ⁄ Weight of sample digested*

*Reference toxicological values*

According to European Regulation EC No 466/2001, the permitted maximum concentration of Cd in fish is 0.05 mg/kg.

### 2.7. Statistical Analyses

Each reported result was the average value of triplicate measurements. Data are presented as min–max values and mean ± standard deviation (SD). The calculations were carried out in EXCEL. The software used for descriptive statistics was SPSS 17.0 for the averages and standard deviations. Because the data were not normally distributed, statistical analyses were performed by non-parametric tests The Student test at a probability of 5% using the section « compare means, one way ANOVA » was used.

## 3. Results and Discussion

### 3.1. Results

The physicochemical parameters of water samples in Aby Lagoon for dry, rainy and flood seasons are presented in Table 2. There is no significant difference between the three seasons. Maximum dissolved oxygen (10.81 mg/L) was recorded in the dry season. The higher value of salinity (6.2) was obtained in the dry season and the lowest (1.5) in the flood season.

**Table 2:** Physicochemical parameters in the Aby lagoon during seasons.

	Dry Season	Rainy Season	Flood Season
**pH**	9.02 ± 0.02	8.5 ± 0.1	9.2 ± 0.1
**T°C**	30.6 ± 0.3	30.9 ± 0.4	31.9 ± 0.1
**Salinity**	6.2 ± 0.4	1.9 ± 0.3	1.5 ±0.1
*DO	10.81 ± 0.02	3.8 ± 0.1	3.04 ± 0.01

*DO: Dissolved oxygen (mg/L).

#### 3.1.1. Cadmium Contamination

**Figure 2:**
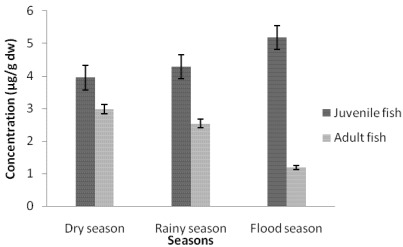
Seasonal variation of cadmium concentrations in muscle tissues of tilapia (*Sarotherodon melanotheron*).

**Figure 3:**
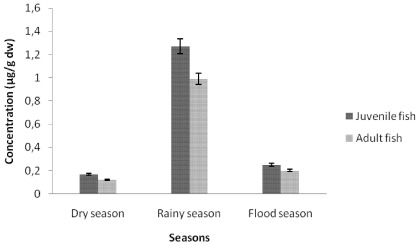
Seasonal variation of cadmium concentrations in liver tissues of tilapia (*Sarotherodon melanotheron*).

**Figure 4:**
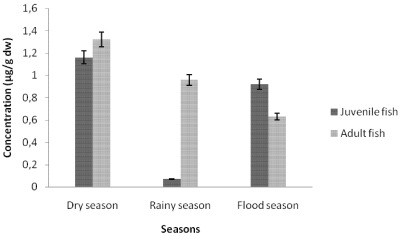
Seasonal variation of cadmium concentrations in gill tissues of tilapia (*Sarotherodon melanotheron*).

The comparison between juvenile and adult fish showed that cadmium concentrations in tissues were not significantly different (*p* value <0.05) during the three studied seasons (Figure 2, Figure 3 and Figure 4). The highest cadmium concentrations were found in muscles of juvenile fish (3.95 ± 1.87, 4.28 ± 3.64, 5.18 ± 2.09 µg/g dw, respectively dry; rainy and flood season). The highest cadmium concentrations in livers (1.27 ± 0.71, 0.99 ± 0.38 µg/g dw, respectively, for juvenile and adult fish) were found during the rainy season, while the highest cadmium concentrations in gills (1.16 ± 0.93, 1.32 ± 0.30 µg/g dw, respectively, in juvenile and adult fish) were found during the dry season. The flood season was characterized by the lowest liver cadmium concentration (0.25 ± 0.11 µg/g dw to 0.20 ± 0.03 µg/g dw). Consequently, the fish tissue contamination by cadmium varied with the season. During the dry season the ranking of cadmium contamination was muscles > gills > livers. During the rainy season the ranking is muscles > livers > gills.

### 3.2. Discussion

The uniformity of water temperature readings may be linked to the shallowness of Aby Lagoon. The dissolved oxygen levels were generally low and below the critical level of 5 mg/L for fish. Lower dissolved oxygen concentration was usually observed at the height of the wet season during which nutrients and debris are flushed into Aby Lagoon with the influx of fresh water from the adjoining rivers (Bia and Tanoe). The months of April to October in West Africa are usually the rainy season. This situation is similar to that reported by McLusky [11] whereby heat generated by sunlight in the dry season months would also cause evaporation of the surface water making it more saline.

The results showed that the cadmium accumulation of muscle tissues in dry (3.95 ± 1.87µg/g dw to 2.98 ± 2.36 µg/g dw), rainy (4.28 ± 3.64 µg/g dw to 2.54 ± 0.97 µg/g dw) and flood (5.18 ± 2.09 µg/g dw to 1.19 ± 0.61 µg/g dw) seasons were relatively higher when compared to European Regulation EC No 466/2001 and FAO/WHO [12] maximum recommended limits of 0.5 mg/kg in fish food. Metal accumulations in fish bodies appear as site specific, corresponding with the metallic toxicity of three aquatic components, viz. water, plankton and sediments [13]. Those heavy metals which form a high proportion of the industrial, municipal and domestic wastes are also found in large proportion in pesticides, fungicides and fertilizers used in agriculture in the Adiake area of South-East Côte d’Ivoire. This situation could also be caused by the mining activities and the lack of environmental regulations.

However Obodai *et al.* [14] and Laar *et al*. [15] found also in *Sarotherodon melanotheron* from Benya Lagoon and Sakumo Lagoon (Ghana) low concentrations of heavy metals below the permissible limits. The levels were also low in comparison to the 0.576–1.257 mg/kg recorded in fishes of Olomoro water bodies [16] and 0.270 mg/kg reported for fishes of the River Niger [17].

Cd profiles recorded in this study were muscle ˃ gills ˃ livers (dry season) and muscle ˃ livers ˃ gills (rainy season). The muscle showed the greatest accumulation of Cd. No similar patterns of Cd accumulation have also been reported in similar studies in fish [6]. Dural *et al*. [18] and Ploetz *et al*. [19] reported highest levels of cadmium in the liver and gills of the fish species *Sparus aurata*, *Dicentrachus labrax*, *Mugil* *cephalus* and *Scomberomorus cavalla*. Yilmaz *et al*. [20] reported that in *Leuciscus cephalus* and *Lepornis gibbosus*, cadmium accumulations in the livers and gills were maximum, while the accumulations were least in fish muscle.

It is important to note that the frequent consumption of *Sarotherodon* in that area could have serious health implications. These findings suggest that heavy metals (such as Cd), which have high affinity for thiol groups, make proteins and peptides susceptible to structural modifications in sub-cellular compartments and tissues as in skeletal muscle. Some authors have already observed that cadmium alters calcium homeostasis [21]. Excessive exposure of human to cadmium can cause death due to its toxicity [22]. It enters cells and accumulates in high concentrations in the cytoplasmic and nuclear space [23]. It has a high affinity for the liver and kidneys [24]. The phenomenon of acute toxicity in humans has been known since 1950 as Itai-Itai syndrome defined by the association of renal failure with osteoporosis (demineralization and weakening of bones) and osteomalacia (demineralization and deformation of bones). It is carcinogenic [25,26] and teratogenic [27]. Genotoxic and apoptosis effects were observed in several types of cells [28].

## 4. Conclusions

*Sarotherodon melanotheron* from Aby Lagoon are fairly contaminated with cadmium and may be toxic to other aquatic fauna and poisonous to human consumers through the trophic web. Results generally showed that cadmium concentrations were highest in the muscle and lowest in the gill and liver. Thus, cadmium pollution seems to be one of the reasons behind the drastic decline of fish observed in Aby Lagoon. Regular monitoring of levels is necessary to assess the impact of heavy metals in this aquatic system.
